# Rapid construction of insulated genetic circuits via synthetic sequence-guided isothermal assembly

**DOI:** 10.1093/nar/gkt860

**Published:** 2013-09-26

**Authors:** Joseph P. Torella, Christian R. Boehm, Florian Lienert, Jan-Hung Chen, Jeffrey C. Way, Pamela A. Silver

**Affiliations:** ^1^Department of Systems Biology, Harvard Medical School, Boston, MA 02115, USA and ^2^Wyss Institute for Biologically Inspired Engineering, Harvard University, Boston, MA 02115, USA

## Abstract

*In vitro* recombination methods have enabled one-step construction of large DNA sequences from multiple parts. Although synthetic biological circuits can in principle be assembled in the same fashion, they typically contain repeated sequence elements such as standard promoters and terminators that interfere with homologous recombination. Here we use a computational approach to design synthetic, biologically inactive unique nucleotide sequences (UNSes) that facilitate accurate ordered assembly. Importantly, our designed UNSes make it possible to assemble parts with repeated terminator and insulator sequences, and thereby create insulated functional genetic circuits in bacteria and mammalian cells. Using UNS-guided assembly to construct repeating promoter-gene-terminator parts, we systematically varied gene expression to optimize production of a deoxychromoviridans biosynthetic pathway in *Escherichia coli*. We then used this system to construct complex eukaryotic AND-logic gates for genomic integration into embryonic stem cells. Construction was performed by using a standardized series of UNS-bearing BioBrick-compatible vectors, which enable modular assembly and facilitate reuse of individual parts. UNS-guided isothermal assembly is broadly applicable to the construction and optimization of genetic circuits and particularly those requiring tight insulation, such as complex biosynthetic pathways, sensors, counters and logic gates.

## INTRODUCTION

Synthetic bacterial pathways and circuits are of great interest for the production of industrial chemicals (1–3) and biofuels (4–7), as well as for biosensing (8,9) and biomedical purposes (10–14). A major goal of synthetic biology is to facilitate these efforts by enabling the assembly of multigene circuits in which each part performs its function predictably, while minimizing unexpected interactions between parts (15,16).

Although such circuits can be assembled one piece at a time (17), serial manipulations are time-consuming, especially when libraries or multiple design-build-test cycles are required to achieve the desired functionality (18). Homologous recombination-based methods such as Gibson isothermal assembly (19) allow the simultaneous assembly of multiple DNA parts and have been used to re-create large natural sequences such as whole genomes (20–22). However, the construction of synthetic circuits can pose additional challenges.

Synthetic circuits are often designed to incorporate standard, insulated parts (15,23,24). Standard parts are desirable because they can be characterized and then easily reused or repurposed between circuits. Insulation, or the degree to which part activity is independent of context or the presence of other parts, is important because it ensures that part activity is consistent between circuits. Owing to the need for standardization and insulation (15,23,24), repeated promoter, ribosome binding site (RBS) and terminator sequences commonly flank the parts comprising synthetic circuits. This is problematic because unique sequences are required for ordered isothermal assembly (19). In principle, one can surmount this by using PCR to flank the repeated sequences with appropriate homology before assembly (25,26), but this can increase both the number of parts and the risk of PCR-based mutations. Alternatively, one can focus on the design of non-repetitive multigene architectures such as operons; however, operons can introduce variation in gene expression and mRNA stability that is not yet fully understood (17,27). Moreover, it is sometimes desirable to independently vary the expression of multiple operons, as demonstrated for taxadiene synthesis in *Escherichia coli* (28).

An alternative approach is to design unique nucleotide sequences (UNSes) to flank each of the parts to be assembled, thereby providing the homology required for ordered assembly of parts containing repeated standard promoter and insulator sequences into functional genetic circuits (26,29,30). Previous work has demonstrated the use of UNSes to assemble multiple parts into functional circuits via recombination-based methods, but not the ability to assemble repetitive sequences or to insulate parts from either the UNSes used or from one another (26). Others have demonstrated some evidence for efficient assembly of repetitive parts, but have not used it to achieve tight insulation or to generate functional genetic pathways (30). Still others have demonstrated assembly of repeating promoter-gene-terminator parts into functional pathways presumed to exhibit some insulation (29). To avoid recombination errors, however, this approach required the use of different promoters and terminators in each part, which can pose a challenge for circuit standardization and scalability.

Here we describe the design of 40-bp UNSes to simultaneously assemble multiple repetitive, well-insulated genetic parts into functional synthetic circuits. We demonstrate that our designed UNSes have minimal unexpected biological activity in bacteria and enable efficient assembly of multiple DNA parts containing repeated promoter, terminator and insulator elements. Using a series of vectors designed to facilitate ligation of UNSes with common standard parts such as BioBricks (31) and BglBricks (32), we used the UNSes to systematically vary expression of multiple well-insulated promoter-gene-terminator units, to construct and optimize a deoxychromoviridans biosynthetic pathway in *E. coli*, and to construct transcriptional AND-logic gates for integration into the genome of embryonic stem cells (33). Our results demonstrate the use of UNS-guided isothermal assembly as a means of constructing and optimizing diverse insulated biological pathways and circuits.

## MATERIALS AND METHODS

### Computational design of UNSes

10^5^ random 40-bp sequences were generated in MATLAB with each nucleotide having an equal chance of being A, T, G or C. This list was systematically reduced by sequentially applying the following design criteria:

1. ATGC Distribution: 45% ≤ GC content ≤55%. No tracts of >4 AT-only or GC-only sequences; 1–2 G/C nucleotides at each terminus.
2. Does not contain start codons (ATG/TTG/CTG). We note that any RBS sequences occurring by chance in the UNSes are predicted to be active only if start codons are close by (34).
3. Does not contain the following common multiple cloning site (MCS) restriction sites: EcoNI, ClaI, XbaI, NcoI, BglII, SpeI, BamHI, NheI, PstI, HindIII, NotI, XhoI, AvrII, BlpI, Bsu36I, AgeI, AflII.
4. Does not contain the following restriction sites commonly used for assembly: AscI, SapI, MauBI, BbsI, MreI, AvrII, BpmI, BsaI.
5. Hairpin T_m_ <40°C assuming 10 mM NaCl and 10 mM Mg^2+^, evaluated with ‘oligoprop’ in MATLAB. Strong hairpins are predicted to be common because of the high Mg^2+^ concentration in isothermal assembly reactions.
6. No bacterial promoter-like sequences identified by PPP (35) or BPROM (SoftBerry).
7. Max score <35.0 when BLASTed against the *E. coli* MG1655 genome.
8. Hybridization of the UNS with all other UNSes has a T_m_ <20°C, evaluated using UNAFold and assuming 100 nM DNA, 10 mM NaCl and 10 mM MgCl_2_.

Supplementary Table S1 lists 10 UNSes designed by this approach and the predicted properties of each. Supplementary Table S2 lists the predicted annealing temperatures for every combination of these 10 UNSes.

### Cloning of part and destination vectors

Basic part and destination vectors were generated by synthesizing a double-stranded gBlock (IDT) fragment containing appropriate UNSes, and inserting it into the desired vector via restriction cloning. BioBrick and conventional restriction cloning were then used to insert promoters, genes and terminators as required. The construction of all part and destination vectors in this work is described in Supplementary Table S3. Primers and gBlocks used to construct these vectors are listed in Supplementary Tables S4 and S5, respectively.

### Digestion, purification and assembly of part and destination vectors

For each assembly, part vectors were digested with one restriction enzyme at the U_N_ site and one restriction enzyme at the U_N+1_ site; the last part was instead digested at the U_N_ and U_X_ sites. Destination vectors were digested at their U_1_ and U_X_ sites. The available restriction sites in each part or destination vector are listed in Supplementary Table S3. Digested destination vectors were PCR-purified using a DNA Clean & Concentrator Kit (Zymo Research), and digested parts were gel-purified using a Gel DNA Recovery Kit (Zymo Research).

Gibson isothermal assembly aliquots were prepared as previously described (19), but the amount of T5 exonuclease was doubled. 100 ng of digested PCR-purified destination vector and equimolar amounts of gel-purified parts were combined in a 5 µl volume, and 5 µl of a 2× isothermal assembly aliquot added. The mixture was incubated in a PCR machine at 50°C for 1 h, with the hot-lid set at 105°C. 2 µl of the assembly mixture was then transformed into TOP10 *E. coli* (Invitrogen).

### Growth and induction

**Figure 1.**
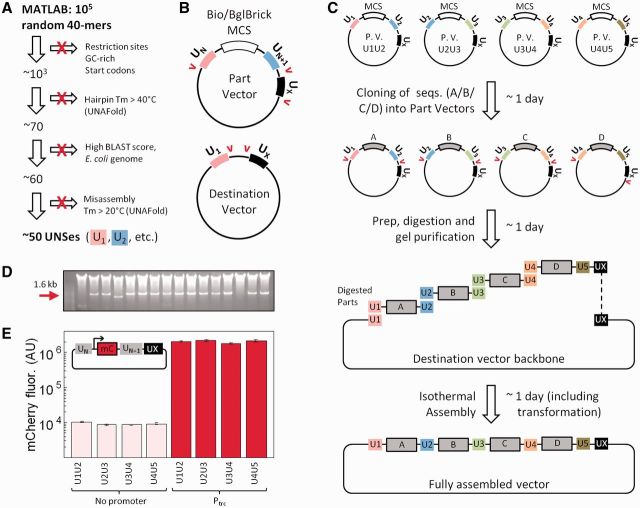
Design and implementation of a synthetic-sequence-guided DNA assembly strategy. (**A**) Computational approach for generating UNSes to facilitate isothermal assembly. In all, 10^5^ random 40-bp sequences were generated in MATLAB, and then eliminated if they contained the indicated sequences (see ‘Materials and Methods’). (**B**) ‘Part’ and ‘destination’ vectors for UNS-guided assembly. Each part vector contains a multiple cloning site (MCS) flanked by two UNSes (e.g. U_1_ + U_2_, U_2_ + U_3_) and a common terminal UNS (U_X_). The MCS contains BioBrick and BglBrick cloning sites. Rare unique restriction sites (red arrows) flank the UNSes. Destination vectors contain only U_1_ and U_X_, and internal restriction sites. (**C**) Diagram of a five-piece assembly, including four part vectors (P.V.) and a destination vector. All part vectors are digested around U_N_ and U_N+1_ except the last, which is digested around U_N_ and U_X_ to permit assembly into U_1_U_X_ of the destination vector. Part vector cloning and assembly into a destination vector takes ∼3 days total. Only ∼1 day is required if the desired part vectors have already been generated. (**D**) Restriction digest of 16 clones from a representative five-piece assembly in which each part was an identical 380-bp sequence. Red arrow indicates the expected 1.6 kb insert. (**E**) Effect of different UNSes on mCherry expression in a part vector with or without a P_trc_ promoter (*N* = 6, error bars = SEM).

**Figure 2.**
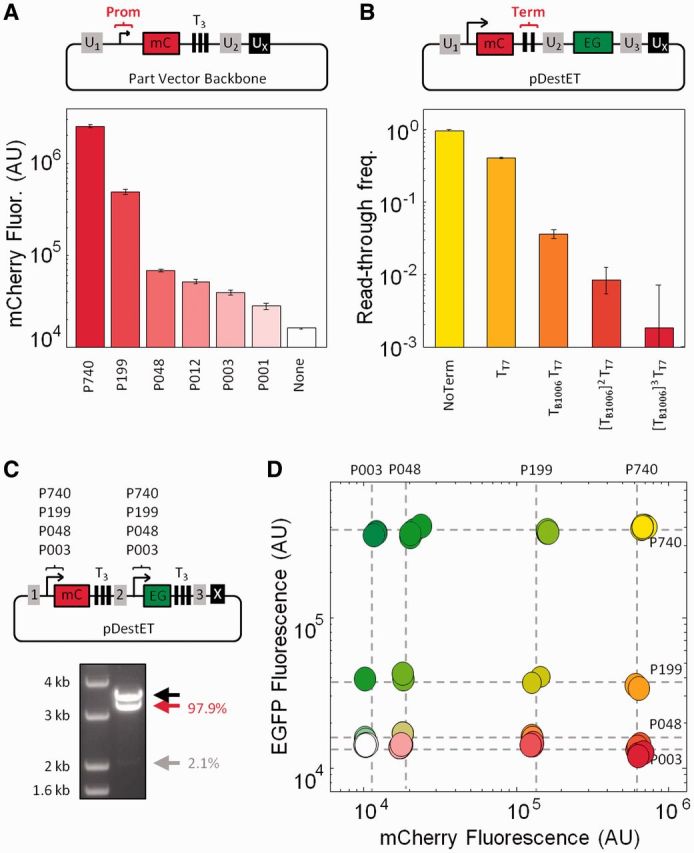
Systematic variation of gene expression via UNS-guided assembly. (**A**) Promoter library. Schematic shows U_1_U_2_ part vector expressing mCherry and indicates the location of the promoter to be varied (red text). The *y*-axis shows mCherry expression from U_1_U_2_ part vectors with different BioFAB promoters; *x*-axis lists the relative strength of each promoter as measured by the BioFAB (*N* = 6, error bars = SEM). (**B**) Terminator testing. Schematic shows the product of assembling a promoter-less U_2_-EGFP-U_3_-U_X_ part vector downstream of a U_1_-P_trc_-mCherry-Term-U_2_ part vector in pDestET. ‘Term’ (red text) represents one of the terminator arrangements listed on the *x*-axis, and is located between mCherry and EGFP in the final construct. Read-through frequency is reported as the ratio of EGFP to mCherry fluorescence, normalized to the no-terminator control (*N* = 6, error bars = SEM). (**C**) Assembly of a fluorescent protein expression library. U_1_U_2_ part vectors contained mCherry, the [T_B1006_]^2^-T_T7_ terminator (T_3_ in figure), and one of four BioFAB promoters. U_2_U_3_ part vectors were the same but contained EGFP. These eight parts were assembled into pDestET, and 60 resulting clones pooled and tested for insert size via restriction digest. Arrows indicate the backbone (black), correct-size insert (red) and a minor incorrect assembly product (gray). In all, 97.9% of inserts are the correct size by densitometry (∼59/60 clones correct). (**D**) Fluorescence of 54 sequenced clones, with the color of each circle indicating a unique set of promoter sequences. Dashed lines indicate the mean mCherry or EGFP fluorescence of all clones with a given promoter sequence; the promoter corresponding to each dashed line is indicated at its intercept.

**Figure 3.**
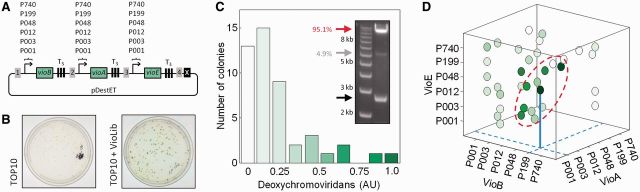
Optimization of deoxychromoviridans production. (**A**) UNS-guided assembly strategy for a promoter library of *vioB*, *vioA* and *vioE*. Each part vector contained a *vio* gene, a [T_B1006_]^2^-T_T7_ terminator (T_3_ in figure) and one of 6 BioFAB promoters. (**B**) TOP10 *E. coli* and TOP10 *E. coli* transformed with the assembled library, grown for 36 h on LB-agar plates. (**C**) Distribution of deoxychromoviridans yields from liquid cultures of individual clones. Yields were measured by extracting deoxychromoviridans from each culture and measuring its absorbance (see ‘Materials and Methods’), normalized to the highest absorbance obtained. Inset: restriction digest of 60 pooled library clones. Arrows indicate the backbone (bottom arrow), expected insert size (top arrow) and a minor, incorrect insert (middle arrow). 95.2% of inserts are the correct size by densitometry (∼57/60 clones correct). (**D**) Plot of individual clones’ deoxychromoviridans production as a function of their promoter strengths. The color of each dot indicates its level of production. The dashed oval highlights a cluster of strains with high production. The highest production strain has medium-to-strong *vioB* and *vioE* expression (P199 and P048, respectively) but weak *vioA* expression (P001). The intersect of the dashed lines indicates this point’s projection onto the vioA–vioB plane, and the solid line connects the point to its projection.

For all expression experiments in Figures 1–3, vectors or isothermal assembly reactions were transformed into TOP10 *E. coli* (Invitrogen) using either the manufacturer's instructions or the TSS competent cell method as previously described (36). Individual colonies were inoculated into 1 ml of LB + 1.0% glucose + 50 µg/ml ampicillin, and grown overnight at 30°C in a 2 ml deep-well plate (Thermo Scientific) with shaking at 1200 rpm on a Titramax 1000 platform shaker (Heidolph). Overnight cultures were diluted 20-fold into 1 ml M9 + 0.5% glucose + 0.1% leucine + 50 μg/ml ampicillin, grown for 4.5 h and induced with 1 mM IPTG for 24 h before analysis.

### Fluorescence and OD_595_ measurements

Twenty-four hours following induction, the OD_595_ from 100 μl of each culture was measured in 200-μl 96-well flat bottom non-treated sterile polystyrene plates (Corning) on a Victor3V 1420 Multilabel Counter (Perkin Elmer) using a 595/60 nm filter. mCherry fluorescence was measured in the same fashion using 565/30 and 630/15 excitation and emission filters, respectively. EGFP fluorescence was measured using 485/14 and 535/25 filters. In both cases, the background due to biomass in each fluorescence channel was corrected for by measuring the mCherry/OD_595_ or EGFP/OD_595_ ratio of wild-type TOP10 cells, multiplying this value by the OD_595_ of each well and subtracting the resulting value from that well’s total fluorescence.

### Extraction and quantification of deoxychromoviridans

Twenty-four hours following induction, the 96-well plates in which the cells were grown were centrifuged for 10 min at 3200 g and their pellets resuspended in 100 µl 10% SDS. The plates were then incubated in a 55°C water bath for 1 h with occasional vortexing. 500 µl ethanol was then added to each well, and the plate incubated at 55°C for 1 h with occasional vortexing. Plates were then left to shake overnight (∼12 h) at 37°C in a Titramax 1000 platform shaker (Heidolph) at 1200 rpm. In the morning, the plates were centrifuged at 3200 g for 10 min, and 100 µl of supernatant transferred to a 200-μl 96-well flat bottom non-treated sterile polystyrene plate (Corning). Each well was then analyzed for absorbance at 650 nm with a Victor3V 1420 Multilabel Counter (Perkin Elmer) using a 650/8 nm filter.

### Analytical digests

**Figure 4.**
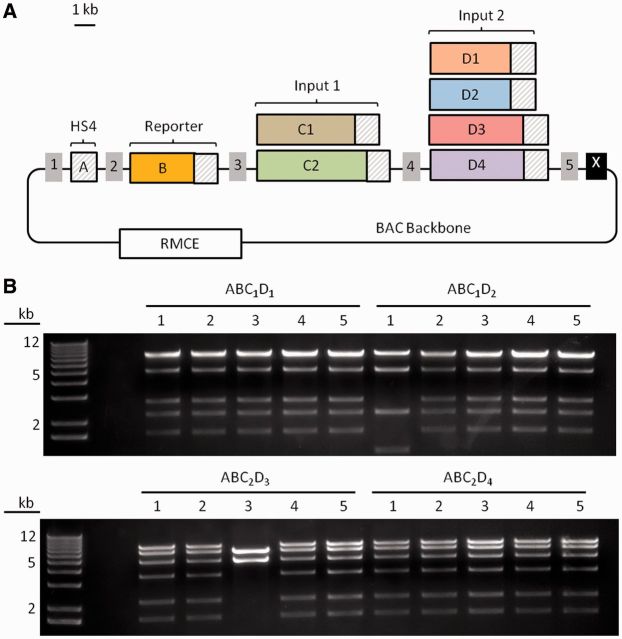
Facile construction of genetic circuits for integration into mammalian chromosomes. (**A**) Construction of an AND gate circuit. Four parts were assembled into pDestRmceBAC, a BAC modified to enable site-specific chromosomal integration in mammalian cells. Parts A and B in all constructs are an HS4 insulator alone and an AND-gated reporter construct plus HS4, respectively. Parts C and D are the two inputs to the AND-gated reporter, but different versions were constructed to verify AND gate functionality; these parts also contain HS4 sequences (striped boxes). (**B**) Analytical restriction digests of assembled AND gate circuit variants with XhoI. Except for ABC_1_D_2_-1 and ABC_2_D_3_-3, all clones yielded the expected digest pattern (18/20 correct).

For the digests in Figure 1, individual clones were mini-prepped (Qiagen) and digested with NsiI and MauBI FastDigest enzymes (Fermentas) to yield expected backbone and insert sizes of 12 308 and 1586 bp, respectively. For the mCherry/EGFP library, 60 clones were pooled, mini-prepped and digested with MluI and Eam1105I FastDigest enzymes to yield expected backbone and insert sizes of 3469 and 3090 bp, respectively. For the *vioBAE* library, 60 clones were pooled, mini-prepped and digested with MluI and BspHI FastDigest enzymes to yield expected backbone and insert sizes of 2584 and 10 052 bp, respectively. Densitometry analysis was carried out using ImageJ’s gel analysis function, and each insert band’s intensity was divided by its length in kilobases before calculating its percentage of the total. We note that densitometry of pooled digested clones can be inaccurate, owing to differences in plasmid yield between clones. In general, plasmid yields between clones varied by <2-fold. The digests in Figure 4 were carried out with XhoI. All restriction digests were run on 1% agarose gels containing ethidium bromide and visualized with 320 nm transillumination.

## RESULTS

We implemented our assembly strategy and demonstrated its use in four steps: (i) we computationally designed 40-bp UNSes and confirmed that they enable efficient assembly of homologous parts, with minimal unwanted biological activity in bacteria; (ii) we demonstrated that parts flanked by UNSes and containing strong termination motifs can be used to systematically vary the expression of multiple fluorescent proteins; (iii) we demonstrated the utility of this system for building and optimizing biosynthetic pathways in *E. coli*, using deoxychromoviridans biosynthesis as a test case; and (iv) we showed how this assembly strategy was used to assemble genetic logic gates for chromosomal integration into embryonic stem cells (33).

### Computational design and performance of UNSes

To design bacterial UNSes for isothermal assembly, we generated 10^5^ random 40-bp sequences and systematically eliminated those that failed to meet two broad criteria: (i) high likelihood of accurate assembly and (ii) low likelihood of affecting nearby gene expression cassettes (Figure 1A and see ‘Materials and Methods’ section). The former was achieved primarily by eliminating poly-GC tracts and high-T_m_ hairpins that may interfere with annealing during isothermal assembly, and by ensuring that different UNSes are unlikely to anneal to one another (see ‘Materials and Methods’ section). The latter was achieved by eliminating UNSes containing start codons, promoter-like sequences or sequences with high BLAST scores against the *E. coli* K12 genome. Only 0.05% of sequences remained after applying these criteria. Ten UNSes and their predicted properties are listed in Supplementary Table S1.

UNSes were used to design a series of ‘part’ and ‘destination’ vectors to facilitate the assembly process (Figure 1B and Supplementary Table S3). The minimal composition of a part vector is: an UNS ‘U_N_’, an MCS with BioBrick cloning sites, a second UNS ‘U_N+1_’ and a third UNS, ‘U_X_’. Although U_N_ and U_N+1_ are different for each vector, U_X_ is common to all part vectors. Desired sequences are cloned into part vectors via conventional methods to, for example, make part vectors bearing promoter-gene-terminator cassettes (Figures 2 and 3); this process can take as little as 1–2 days, including plasmid isolation and restriction digest- or PCR-based confirmation of successful cloning. To assemble multiple part vectors into a single construct, each is digested with restriction sites flanking U_N_ and U_N+1_ except for the final part in the series, which is instead digested at restriction sites flanking U_N_ and U_X_ (Supplementary Table S3). The resulting UNS-flanked fragments are gel-purified and assembled into a digested PCR-purified destination vector via isothermal assembly. Destination vectors contain UNSes U_1_ and U_X_, and therefore contain homology to the first and last of the parts (Figure 1C and see ‘Materials and Methods’ section). From part vector cloning to assembly, this process can take as little as 2–3 days (Figure 1C). If desired part vectors already exist, this process can take as little as 1 day.

Consistent with our design, parts flanked by computationally designed UNSes assembled efficiently even when they contained substantial sequence homology. We first performed a 5-piece assembly of digested part vectors, in which each vector contained only a T7 promoter, MCS and T7 terminator (pJT170, 172, 174, 176, Supplementary Table S3), into the destination vector pDestBAC (Supplementary Table S3 and Figure 1C). Each digested part was ∼80% identical, with the UNSes providing the only differences in sequence. Following transformation into TOP10 *E. coli*, ∼85% of clones reproducibly contained correctly sized inserts; analytical digests from a representative experiment are shown in Figure 1D. Sequencing showed that all correctly sized inserts had the expected sequence, whereas incorrectly sized inserts were due to recombination between non-sequential parts (e.g. U_1_U_2_ and U_4_U_5_). All other assembly reactions in this work, in which parts had substantially <80% sequence homology, assembled correctly 90–98% of the time (Figures 2–4).

Also consistent with our design strategy, UNSes showed minimal biological activity in bacteria as measured by their effects on proximal promoter cassettes. We cloned mCherry with either no promoter or a strong (P_trc_) promoter, into each of four part vectors bearing different UNSes (pJT170, 172, 174, 176; Supplementary Table S3), and measured the resulting fluorescence in TOP10 *E. coli* (Figure 1E). Although the P_trc_ vectors produced about 100-fold more mCherry than their promoter-less counterparts, variation within the promoter-less vectors or P_trc_ vectors was minimal (<18% difference in mean fluorescence). This indicated that the UNSes did not differ substantially in their effects on proximal expression cassettes.

### Systematic variation of gene expression using UNS-guided isothermal assembly

Rational optimization of biological circuits requires a way to systematically and independently vary the expression of multiple genes. We asked whether UNS-guided assembly could be used to construct expression libraries of multiple strongly insulated parts, such that the promoter strength of a part would determine only its own expression level, without affecting others.

We first chose a small set of promoters from the BioFAB library (37) and cloned them into a U_1_-mCherry-U_2_ part vector (pJT260; Supplementary Table S3), resulting in variation of promoter strength over a 100-fold range (Figure 2A). To ensure translation rates were not affected by our choice of promoter, we chose only those promoters that were identical downstream of their transcription start sites, and therefore would not alter mRNA 5′ UTRs (38).

We also built a set of terminator variants and identified those sufficient to insulate our promoter library (Figure 2B). Terminator variants consisted of a mixture of T7 terminators and repeats of BBa_B1006, a strong terminator BioBrick (39), and were cloned into a U_1_-P_trc_-mCherry-U_2_ part vector (pJT257, 260, 318, 320, 321; Supplementary Table S3). Read-through frequency was assessed by assembling each part upstream of U_2_-EGFP-U_3_-U_X_ (pJT345; Supplementary Table S3), in the multicopy destination vector pDestET (Supplementary Table S3), and measuring the ratio of EGFP to mCherry fluorescence (see ‘Materials and Methods’ section and Figure 2B). The triple terminator ([T_B1006_]^2^-T_T7_) decreased read-through by ∼100-fold, and was therefore the smallest terminator sufficient to insulate members of the promoter library from one another.

Combining the promoter library and terminators with our UNS-guided assembly strategy, we were able to independently titrate the expression of two fluorescent proteins. Using restriction cloning, we generated part vectors with U_1_-P_trc_-mCherry-[T_B1006_]^2^-T_T7_ -U_2_ (pJT260; Supplementary Table S3) and U_2_-P_trc_-EGFP-[T_B1006_]^2^-T_T7_-U_3_-U_X_ (pJT336, Supplementary Table S3), cloned four of the BioFAB promoters in place of each part vector’s P_trc_ promoter and assembled the resulting parts into pDestET (Figure 2C) to generate a small library of 16 fluorescent protein expression variants. Sixty of the resulting clones were sequenced, and tested for insert size, OD_595_, and mCherry and EGFP fluorescence (see ‘Materials and Methods’ section). Despite the presence of homology-rich triple-terminators in each part, nearly all clones assembled correctly (Figure 2C; in all, 97.9% of inserts were the correct size by densitometry, or ∼59/60 clones, and at least 54/60 clones contained expected promoter upstream regions as evaluated by sequencing). A plot of mCherry versus EGFP fluorescence (Figure 2D) showed that expression of mCherry could be titrated over a ∼100-fold expression range without substantially affecting EGFP expression. The reverse was also true, suggesting that strong insulation is provided by the [T_B1006_]^2^-T_T7_ terminators. These results demonstrated that UNS-guided assembly makes it possible to efficiently construct tightly insulated multigene circuits despite the presence of repeated sequence elements.

### Optimization of deoxychromoviridans production in *E. coli*

The ability to assemble multiple well-insulated parts should enable rational optimization of genetic circuits. As a test case for UNS-based optimization of a bacterial biosynthetic pathway, we chose three genes from *Chromobacterium violaceum*, *vioB*, *vioA* and *vioE*, which together are capable of catalyzing the conversion of tryptophan to prodeoxyviolacein (40). In *C.violaceum*, the genes *vioC* and *vioD* normally convert prodeoxyviolacein to violacein, a compound of interest due to its antibiotic and antitumor properties (41); in their absence, however, prodeoxyviolacein undergoes spontaneous oxidation and dimerization to produce an insoluble green compound, deoxychromoviridans (40). The absorbance of deoxychromoviridans, therefore, provides a simple assay for the activity of the first three steps in the violacein pathway (42).

To optimize deoxychromoviridans production, we used the same strategy as in Figure 2 to vary the expression of *vioB*, *vioA* and *vioE* independently. We built part vectors containing each of the three genes and a [T_B1006_]^2^-T_T7_ terminator (pJT369, 371, 375; Supplementary Table S3), then cloned 6 BioFAB promoters into each. The resulting 18 parts were assembled into pDestET (Figure 3A) to generate a 216-member pathway library and transformed into TOP10 *E. coli*, yielding colonies with clear differences in pigmentation (Figure 3B). Sixty colonies were chosen at random, sequenced, and tested for insert size, OD_595_ and deoxychromoviridans production by A650 (see ‘Materials and Methods’). The pathway assembled efficiently (Figure 3C; in all, 95.2% of inserts were the correct size by densitometry or ∼57/60 clones, and at least 52/60 clones contained expected promoter upstream regions as evaluated by sequencing), and the chosen clones exhibited a wide range of deoxychromoviridans yields in liquid culture (Figure 3C).

Analysis of deoxychromoviridans production by these clones showed a distribution of yields that varied according to the strengths of their promoters (Figure 3D). High-production strains clustered in a region of expression space with low expression of VioA and moderate expression of VioB and VioE (Figure 3D). This local optimum indicated that strong expression of all three genes—a typical first approach to the design of novel metabolic pathways—was not an optimal design strategy. Moreover, it suggested that strong expression of *vioA* may be toxic; indeed, the two *vioA* part vectors with the strongest promoters had small colony sizes compared with the lower promoter strength VioA parts (data not shown). These results demonstrated the potential of UNS-guided assembly to identify local production optima in gene expression space and to identify pathway features that may limit activity.

### Assembly of AND-logic gates for genomic integration into mammalian stem cells

Mammalian genetic circuits are of interest due to their potential for analytical, therapeutic and diagnostic applications (8,11), but they are challenging to assemble and, for most practical applications, must be integrated into the genome. We previously used UNS-guided assembly to assemble two- and three-input split-TALE AND gate circuits into a single bacterial artificial chromosome (BAC) engineered to facilitate mammalian chromosomal integration (33). Here we present the construction methodology used to generate those circuits.

Figure 4 shows the approach taken to construct several AND gate variants in BACs designed to facilitate chromosomal integration. Variants of the requisite four parts were first cloned into part vectors (pFL part vectors; Supplementary Table S3), then assembled in different combinations into pDestRmceBAC (Supplementary Table S3), which carries the sequences required for recombination-mediated cassette exchange (RMCE)-based single-copy integration (43, 44) (Figure 4A). In all constructs, parts A and B contained an HS4 insulator sequence (45) and an AND-gated reporter construct, respectively. Parts C and D contained the two inputs to the AND-gated reporter, and different versions of the AND gate were assembled with different versions of C and D. For instance, parts D1 and D2 encoded one of the two AND gate inputs, but D2 contained a loss of function mutation; AND gates constructed with D_2_ could, therefore, be used as a negative control for AND gates constructed with D_1_. Likewise, other part variants helped confirm that the AND gate behaved as expected (33). It is worth noting that all parts contained identical HS4 insulator sequences at their termini to minimize undesirable part interactions, and therefore required UNSes for assembly.

Analytical restriction digests of 20 clones from 4 unique AND gate assemblies showed that 18 (90%) of the clones had assembled successfully (Figure 4B). Site-specific integration of correctly assembled constructs into mouse embryonic stem cells was then carried out (33). We note that some AND gates were also assembled into BACs containing the PiggyBac transposase (Supplementary Table S3), allowing random but highly efficient integration into the genome (46). As part vectors can easily be modified and reassembled, UNS-guided assembly is an attractive approach for the rapid prototyping, modification and integration of genetic circuits into mammalian cells.

## DISCUSSION

In this work, we computationally designed 40-bp UNSes and used them to perform isothermal assembly of multipart bacterial metabolic pathways and mammalian genetic circuits. Importantly, we demonstrated that our designed UNSes did not substantially affect local bacterial promoters, and enabled tight insulation by facilitating the assembly of parts with repeated sequences such as terminators and HS4 insulators. Insulation is an important design principle in synthetic biology and greatly simplifies circuit design, as well-insulated parts have predictable functions and can be assembled in a modular way to rationally modify existing circuits or to build new ones (15).

To facilitate assembly, we also generated a series of standard part and destination vectors so that individual parts could be easily modified and reused. Standardization is important because it greatly reduces the time and effort required to modify, optimize or repurpose existing circuits for novel applications (24). We also designed our part vectors to facilitate integration with BioBrick and BglBrick standards, as a wide range of Bricks are publicly available, but these generally cannot be used for simultaneous multipart assembly.

We note that although methods based on type II S restriction sites (47) or PCR (48,49) also enable simultaneous assembly of multiple parts, the former is commonly found in natural coding sequences, and the latter can introduce point mutations, both of which pose challenges for the construction of large circuits (30).

During preparation of this article, work was published demonstrating a facile UNS-based assembly strategy for mammalian genes, in which multiple fluorescent reporters were assembled as a proof of principle (30). Our study extends this work by demonstrating that appropriately designed UNSes do not affect nearby expression cassettes, that they can be used to assemble well-insulated parts with repeated terminator or insulator sequences, and that this approach can be used to titrate gene expression levels in a modular way (Figure 2). We also demonstrate the use of our method to efficiently construct and optimize insulated functional circuits such as biosynthetic pathways (Figure 3) and transcriptional logic gates (33) (Figure 4). Future efforts to systematically test and identify UNS design principles will be valuable in further improving assembly efficiency and insulation, and may enable the construction of more complex pathways and genetic circuits.

We optimized deoxychromoviridans biosynthesis in *E. coli* by using UNS-guided assembly to vary multiple promoter strengths. This approach is attractive because it can identify design principles by which to improve yield in the next round of assembly (17). As an example, our results suggested that too much expression of VioA, the first enzyme in the pathway from tryptophan to deoxychromoviridans, is toxic. This toxicity may be due to protein burden, to VioA rapidly depleting cellular tryptophan, or because the product of VioA, IPA imine (40), is itself toxic. This information can then be integrated into predictive models to rationally improve biosynthetic pathways or genetic circuits (50). In principle, one can also use UNSes to experimentally find local optima in expression space, for instance by modifying and re-assembling part vectors with increasingly narrow ranges of promoter strengths. Given the rapid pace at which part vectors can be modified and reassembled (Figure 1C), such design-build-test cycles could be completed as frequently as twice per week. By including additional part vectors in subsequent assemblies, existing circuits can also be repurposed for more complex applications.

We also used UNSes to construct mammalian transcriptional AND gates with repeating HS4 insulation sequences (Figure 4). These were assembled in BACs modified to facilitate site-specific genomic integration, and used both to verify AND gate function and to optimize their performance when integrated into the genome of embryonic stem cells (33). Our approach is therefore attractive as a means of speeding the design-build-test cycle of genomically integrated synthetic circuits, which have potential in diagnostic and therapeutic applications (8,11,12).

The methodology we describe is straightforward, efficient and modular. It permits assembly of repetitive, tightly insulated parts and can be easily adapted to diverse genetic engineering applications. Part and destination vectors designed for various bacterial applications have been constructed (Supplementary Table S3), as are destination vectors for mammalian cell transfection, transposition and site-specific integration (33) (Supplementary Table S3). Moreover, the sequences required to generate new part and destination vectors are small enough to be synthesized at low cost (<200 bp). As UNS-guided assembly can speed the design-build-test cycle for complex circuits with repeated promoters, terminators and insulators, we anticipate application of this approach to a wide range of applications where multigene assembly and insulation are desired. These include the construction and optimization of complex metabolic pathways, the development of genetic timers and counters and the construction of multi-input logic gates.

## SUPPLEMENTARY DATA

Supplementary Data are available at NAR Online.

## FUNDING

Advanced Research Projects Agency-Energy ‘Electrofuels' Collaborative Agreement [DE-AR0000079 to P.A.S.]; National Science Foundation Graduate Research Fellowship and Herchel Smith Graduate Research Fellowship (to J.P.T.); German National Academic Foundation Scholarship (to C.R.B.); European Molecular Biology Organization and Human Frontier Science Program Fellowship (to F.L.); Natural Sciences and Engineering Research Council of Canada Postdoctoral Fellowship (to J.H.C.); Defense Advanced Research Projects Agency [4500000572 to P.A.S.]. This material is based upon work supported by the National Science Foundation. Any opinions, findings and conclusions or recommendations expressed in this material are those of the authors and do not necessarily reflect the views of the National Science Foundation. Funding for open access: Advanced Research Projects Agency-Energy ‘Electrofuels' Collaborative Agreement [DE-AR0000079 to P.A.S.].

*Conflict of interest statement*. None declared.

## Supplementary Material

Supplementary Data
