# Supplementary material for: Rapid construction of insulated genetic circuits via synthetic sequence-guided isothermal assembly
Source: Nucleic Acids Res. 2013 Sep 26;42(1):681–9. doi: 10.1093/nar/gkt860 (PMC3874176; doi:10.1093/nar/gkt860)
Supplement: Supplementary Data [file supp_42_1_681__index.html]

Rapid construction of insulated genetic circuits via synthetic sequence-guided isothermal assembly — Rapid construction of insulated genetic circuits via synthetic sequence-guided isothermal assembly — Supplementary Data 

# Rapid construction of insulated genetic circuits via synthetic sequence-guided isothermal assembly

## Supplementary Data

files

**Files in this Data Supplement:**

- Supplementary Data - pdf file
