# Supplementary material for: Rapid construction of insulated genetic circuits via synthetic sequence-guided isothermal assembly
Source: Nucleic Acids Res. 2013 Sep 26;42(1):681–9. doi: 10.1093/nar/gkt860 (PMC3874176; doi:10.1093/nar/gkt860)
Supplement: Supplementary Data [file supp_gkt860_nar-02242-h-2013-File006.pdf]

SUPPORTING INFORMATION

Table S1. 10 designed UNS sequences and their properties

UNS#	Sequence	GC%	No AT/GC tracts*	Ends with C/G*	No Start Codons*	Restriction sites OK*	Hairpin Max Tm (°C)*	Lacks promoter-like sequences*	Max BLAST Score*
1	CATTACTCGCATCCATTCTCAGGCTGTCTCGTCTCGTCTC	52.5	✓	✓	✓	✓	27.1	✓	26.3
2	GCTGGGAGTTCGTAGACGGAAACAAACGCAGAATCCAAGC	52.5	✓	✓	✓	✓	39.2	✓	32.2
3	GCACTGAAGGTCCTCAATCGCACTGGAAACATCAAGGTCG	52.5	✓	✓	✓	✓	33.9	✓	26.3
4	CTGACCTCCTGCCAGCAATAGTAAGACAACACGCAAAGTC	50.0	✓	✓	✓	✓	33.5	✓	28.2
5	GAGCCAACTCCCTTTACAACCTCACTCAAGTCCGTTAGAG	50.0	✓	✓	✓	✓	38.5	✓	26.3
6	CTCGTTCGCTGCCACCTAAGAATACTCTACGGTCACATAC	50.0	✓	✓	✓	✓	36.6	✓	26.3
7	CAAGACGCTGGCTCTGACATTTCCGCTACTGAACTACTCG	52.5	✓	✓	✓	✓	26	✓	26.3
8	CCTCGTCTCAACCAAAGCAATCAACCCATCAACCACCTGG	52.5	✓	✓	✓	✓	39	✓	30.2
X	CCAGGATACATAGATTACCACAACTCCGAGCCCTTCCACC	52.5	✓	✓	✓	✓	30.7	✓	24.3
9	GTTCCTTATCATCTGGCGAATCGGACCCACAAGAGCACTG	52.5	✓	✓	✓	✓	30.8	✓	28.2

* See Methods in main text for specific details of each category.

Table S2. Predicted hybridization Tm (°C) of UNS pairs

	1	2	3	4	5	6	7	8	X	9
1	81									
2	-5	79								
3	-5	0	79							
4	-5	-5	4	79						
5	-5	-5	-5	-5	79					
6	-5	-5	-5	-5	-5	78				
7	5	-5	1	-5	-5	7	80			
8	-5	-5	-2	3	6	-5	-5	79		
X	-5	-5	-5	-5	-5	-5	-5	16	78	
9	-5	-5	18	-5	-5	-5	-2	18	17	81

Table S3. Part and destination vectors used in this work

**Basic Part vectors**							
**Plasmid**	**Part Contains:**	**Construction**	**Resistance**	**Copy Number**	**UN Digest Sites**	**UN+1 Digest Sites**	**UX Digest Sites**
pFL_U1U2	U1-MCS-U2-UX	MfeI/NsiI digested gBlock_U1U2_FL was ligated into EcoRI/PstI digested BioBrick-modified pIDTSmart vector (IDT).	Amp	~500	AscI/BspMI	MauBI/BbsI	MreI/BsaI
pFL_U2U3	U2-MCS-U3-UX	MfeI/NsiI digested gBlock_U2U3_FL was ligated into EcoRI/PstI digested BioBrick-modified pIDTSmart vector (IDT).	Amp	~500	AscI/BspMI	MauBI/BbsI	MreI/BsaI
pFL_U3U4	U3-MCS-U4-UX	MfeI/NsiI digested gBlock_U3U4_FL was ligated into EcoRI/PstI digested BioBrick-modified pIDTSmart vector (IDT).	Amp	~500	AscI/BspMI	MauBI/BbsI	MreI/BsaI
pFL_U4U5	U4-MCS-U5-UX	MfeI/NsiI digested gBlock_U4U5_FL was ligated into EcoRI/PstI digested BioBrick-modified pIDTSmart vector (IDT).	Amp	~500	AscI/BspMI	MauBI/BbsI	MreI/BsaI
pJT170	U1-PT7-MCS-TT7-U2-UX	pETDuet-1 digested with XbaI, blunted with klenow fragment, and digested again with DraIII, followed by ligation with phosphorylated, DraIII-digested gBlockU1U2. SphI/MfeI then digested, blunted, and re-ligated.	Amp	~40	AflII/SapI	MauBI/BbsI	MreI/BsaI
pJT172	U2-PT7-MCS-TT7-U3-UX	pETDuet-1 digested with XbaI, blunted with klenow fragment, and digested again with DraIII, followed by ligation with phosphorylated, DraIII-digested gBlockU2U3. SphI/MfeI then digested, blunted, and re-ligated.	Amp	~40	AflII/SapI	MauBI/BbsI	MreI/BsaI
pJT174	U3-PT7-MCS-TT7-U4-UX	pETDuet-1 digested with XbaI, blunted with klenow fragment, and digested again with DraIII, followed by ligation with phosphorylated, DraIII-digested gBlockU3U4. SphI/MfeI then digested, blunted, and re-ligated.	Amp	~40	AflII/SapI	MauBI/BbsI	MreI/BsaI
pJT176	U4-PT7-MCS-TT7-U5-UX	pETDuet-1 digested with XbaI, blunted with klenow fragment, and digested again with DraIII, followed by ligation with phosphorylated, DraIII-digested gBlockU4U5. SphI/MfeI then digested, blunted, and re-ligated.	Amp	~40	AflII/SapI	MauBI/BbsI	MreI/BsaI
pJT260	U1-Ptrc-mCherry-[TB1006]^2-TT7-U2-UX	Described below.	Amp	~40	AflII/SapI	MauBI/BbsI	MreI/BsaI
pJT288	U2-Ptrc-mCherry-[TB1006]^2-TT7-U3-UX	ClaI/XhoI fragment of pJT260 cloned into ClaI/XhoI-digested pJT172.	Amp	~40	AflII/SapI	MauBI/BbsI	MreI/BsaI
pJT290	U3-Ptrc-mCherry-[TB1006]^2-TT7-U4-UX	ClaI/XhoI fragment of pJT260 cloned into ClaI/XhoI-digested pJT174.	Amp	~40	AflII/SapI	MauBI/BbsI	MreI/BsaI
pJT292	U4-Ptrc-mCherry-[TB1006]^2-TT7-U5-UX	ClaI/XhoI fragment of pJT260 cloned into ClaI/XhoI-digested pJT176.	Amp	~40	AflII/SapI	MauBI/BbsI	MreI/BsaI
**Basic Destination vectors**							
**Plasmid**	**Destination UNSes & Backbone:**	**Construction**	**Resistance**	**Copy Number**	**U1 Digest Sites**	**UX Digest Sites**	
pDestET	U1-UX. Backbone: pETDuet-1 (Novagen) lacking promoter, MCS, T7Term and F1 Ori.	gBlock_U1UX_A digested with SphI/XbaI and ligated into SphI/AvrII-digested pETDuet-1.Resulting plasmid then digested with BlpI/SspI to eliminate F1 Ori, blunted with Klenow fragment, and re-ligated.	Amp	~40	MauBI/BbsI	AvrII/BpmI	
pDestBAC	U1-UX. Backbone: pETCoco-1 (Novagen) with stop codon at NheI site.	pETCoCo-1 digested with NheI, blunted with KF, and re-ligated to generate a stop codon. Resulting plasmid then digested with HindIII/AvrII and HindIII/XbaI digested gBlock_U1UX_B ligated in.	Cam	~1 (Amplifiable to 40 with arabinose)	MauBI/BbsI	AvrII/BpmI	

pDestRmce BAC	U1-UX. Backbone: pETCoco-1 containing cassette for RMCE.	Multiple steps. In short, the T7 Promoter, MCS and LoxP site from pETCoco-1 were removed and replaced with a U1-UX cassette and the inverted LoxP sites required for RMCE-based integration (Ref. #33 in main text).	Cam	~1 (Amplifiable to 40 with arabinose)	MauBI/BbsI	AvrII/BpmI	
pDestPB BAC	U1-UX. Backbone: pETCoco-1 containing cassette for PiggyBAC transposition.	Multiple steps. In short, the T7 Promoter and MCS from pETCoco-1 were removed and replaced with inverted terminal repeats (ITRs) containing the U1-UX cassette, HS4 and a blasticidin resistance gene. The PiggyBAC transposase was cloned outside of the ITRs. (Ref. #33 in main text).	Cam	~1 (Amplifiable to 40 with arabinose)	MauBI/BbsI	AvrII/BpmI	
**Part vectors for terminator testing**							
**Plasmid**	**Part Contains:**	**Construction**	**Resistance**	**Copy Number**	**UN Digest Site**	**UN+1 Digest Site**	**UX Digest Site**
pJT233	U1-PT7-mCherry-TT7-U2-UX	NcoI/XbaI mCherry fragment of pDHL374 ligated into NcoI/SpeI-digested pJT170.	Amp	~40	AflII/SapI	MauBI/BbsI	MreI/BsaI
pJT246	U1-Ptrc-mCherry-TT7-U2-UX	Ptrc fragments were generated by annealing primers JT326 and JT328, filling in the gaps with Klenow fragment, digesting with ClaI/XbaI, and ligating into ClaI/XbaI-digested pJT233.	Amp	~40	AflII/SapI	MauBI/BbsI	MreI/BsaI
pJT257	U1-Ptrc-mCherry-TB1006-TT7-U2-UX	BBa_B1006 terminator fragments were generated by annealing phosphorylated primers JT356 and JT357, and ligating into NheI/PstI-digested pJT246.	Amp	~40	AflII/SapI	MauBI/BbsI	MreI/BsaI
pJT260	U1-Ptrc-mCherry-[TB1006]^2-TT7-U2-UX	BBa_B1006 terminator fragment ligated into SpeI/PstI-digested pJT257.	Amp	~40	AflII/SapI	MauBI/BbsI	MreI/BsaI
pJT321	U1-Ptrc-mCherry-[TB1006]^3-TT7-U2-UX	BBa_B1006 terminator fragment ligated into SpeI/PstI-digested pJT260.	Amp	~40	AflII/SapI	MauBI/BbsI	MreI/BsaI
pJT318	U1-Ptrc-mCherry-U2-UX	pJT260 digested with BamHI/AgeI, blunted with klenow fragment, and re-ligated to remove all terminators.	Amp	~40	AflII/SapI	MauBI/BbsI	MreI/BsaI
pJT320	U1-Ptrc-mCherry-TT7-U2-UX	pJT260 digested with BamHI/SpeI, blunted with klenow fragment, and re-ligated to remove all but the T7term.	Amp	~40	AflII/SapI	MauBI/BbsI	MreI/BsaI
pJT241	U2-PT7-EGFP-TT7-U3-UX	NcoI/XbaI EGFP fragment from pEGFP (Clontech) ligated into NcoI/SpeI-digested pJT172.	Amp	~40	AflII/SapI	MauBI/BbsI	MreI/BsaI
pJT323	U1-Ptrc-EGFP-[TB1006]^2-TT7-U2-UX	NcoI/BamHI EGFP fragment of pJT241 ligated into NcoI/BamHI-digested pJT260.	Amp	~40	AflII/SapI	MauBI/BbsI	MreI/BsaI
pJT336	U2-Ptrc-EGFP-[TB1006]^2-TT7-U3-UX	XbaI/XhoI fragment of pJT323 ligated into XbaI/XhoI-digested pJT288.	Amp	~40	AflII/SapI	MauBI/BbsI	MreI/BsaI
pJT345	U2-EGFP-[TB1006]^2-TT7-U3-UX	pJT336 digested with ClaI/XbaI, blunted with klenow fragment, and re-ligated to remove the promoter.	Amp	~40	AflII/SapI	MauBI/BbsI	MreI/BsaI
**Part vectors for testing UNS effects on proximal expression cassettes**							
**Plasmid**	**Part Contains:**	**Construction**	**Resistance**	**Copy Number**	**UN Digest Site**	**UN+1 Digest Site**	**UX Digest Site**
pJT316	U1-mCherry-[TB1006]^2-TT7-U2-UX	pJT260 digested with ClaI/XbaI, blunted with klenow fragment, and re-ligated to remove the promoter.	Amp	~40	AflII/SapI	MauBI/BbsI	MreI/BsaI
pJT339	U2-mCherry-[TB1006]^2-TT7-U3-UX	pJT288 digested with ClaI/XbaI, blunted with klenow fragment, and re-ligated to remove the promoter.	Amp	~40	AflII/SapI	MauBI/BbsI	MreI/BsaI
pJT341	U3-mCherry-[TB1006]^2-TT7-U4-UX	pJT290 digested with ClaI/XbaI, blunted with klenow fragment, and re-ligated to remove the promoter.	Amp	~40	AflII/SapI	MauBI/BbsI	MreI/BsaI

pJT343	U4-mCherry-[TB1006]^2-TT7-U5-UX	pJT292 digested with ClaI/XbaI, blunted with klenow fragment, and re-ligated to remove the promoter.	Amp	~40	AflII/SapI	MauBI/BbsI	MreI/BsaI
pJT260	U1-Ptrc-mCherry-[TB1006]^2-TT7-U2-UX	Described above	Amp	~40	AflII/SapI	MauBI/BbsI	MreI/BsaI
pJT288	U2-Ptrc-mCherry-[TB1006]^2-TT7-U3-UX	Described above	Amp	~40	AflII/SapI	MauBI/BbsI	MreI/BsaI
pJT290	U3-Ptrc-mCherry-[TB1006]^2-TT7-U4-UX	Described above	Amp	~40	AflII/SapI	MauBI/BbsI	MreI/BsaI
pJT292	U4-Ptrc-mCherry-[TB1006]^2-TT7-U5-UX	Described above	Amp	~40	AflII/SapI	MauBI/BbsI	MreI/BsaI
**Part vectors for promoter titration and mCherry/EGFP library construction**							
**Plasmid**	**Part Contains:**	**Construction**	**Resistance**	**Copy Number**	**UN Digest Site**	**UN+1 Digest Site**	**UX Digest Site**
pJT301	U1-P740-mCherry-[TB1006]^2-TT7-U2-UX	Primers JT407/408 phosphorylated, annealed and ligated into ClaI/XbaI-digested pJT260.	Amp	~40	AflII/SapI	MauBI/BbsI	MreI/BsaI
pJT303	U1-P199-mCherry-[TB1006]^2-TT7-U2-UX	Primers JT409/410 phosphorylated, annealed and ligated into ClaI/XbaI-digested pJT260.	Amp	~40	AflII/SapI	MauBI/BbsI	MreI/BsaI
pJT305	U1-P048-mCherry-[TB1006]^2-TT7-U2-UX	Primers JT411/412 phosphorylated, annealed and ligated into ClaI/XbaI-digested pJT260.	Amp	~40	AflII/SapI	MauBI/BbsI	MreI/BsaI
pJT309	U1-P003-mCherry-[TB1006]^2-TT7-U2-UX	Primers JT415/416 phosphorylated, annealed and ligated into ClaI/XbaI-digested pJT260.	Amp	~40	AflII/SapI	MauBI/BbsI	MreI/BsaI
pJT353	U2-P740-EGFP-[TB1006]^2-TT7-U3-UX	Primers JT407/408 phosphorylated, annealed and ligated into ClaI/XbaI-digested pJT336.	Amp	~40	AflII/SapI	MauBI/BbsI	MreI/BsaI
pJT351	U2-P199-EGFP-[TB1006]^2-TT7-U3-UX	Primers JT409/410 phosphorylated, annealed and ligated into ClaI/XbaI-digested pJT336.	Amp	~40	AflII/SapI	MauBI/BbsI	MreI/BsaI
pJT349	U2-P048-EGFP-[TB1006]^2-TT7-U3-UX	Primers JT411/412 phosphorylated, annealed and ligated into ClaI/XbaI-digested pJT336.	Amp	~40	AflII/SapI	MauBI/BbsI	MreI/BsaI
pJT347	U2-P003-EGFP-[TB1006]^2-TT7-U3-UX	Primers JT415/416 phosphorylated, annealed and ligated into ClaI/XbaI-digested pJT336.	Amp	~40	AflII/SapI	MauBI/BbsI	MreI/BsaI
**Deoxychromoviridans part vectors**							
**Plasmid**	**Part Contains:**	**Construction**	**Resistance**	**Copy Number**	**UN Digest Site**	**UN+1 Digest Site**	**UX Digest Site**
pJT369	U1-P003-VioB-[TB1006]^2-TT7-U2-UX	Primers JT446/447 phosphorylated, annealed and ligated into pVioB to remove internal XbaI site. XbaI-SspI fragment of resulting vector then cloned into XbaI/StuI-digested pJT309.	Amp	~40	AflII/SapI	MauBI/BbsI	MreI/BsaI
pJT375	U2-P003-VioA-[TB1006]^2-TT7-U3-UX	XbaI-BsaAI fragment of pVioA ligated into BamHI-BLUNT/XbaI digested pJT347.	Amp	~40	AflII/SapI	MauBI/BbsI	MreI/BsaI
pJT371	U3-Ptrc-VioE-[TB1006]^2-TT7-U4-UX	XbaI-BsaAI fragment of pVioE ligated into XbaI/EcoRV-digested pJT290.	Amp	~40	AflII/SapI	MauBI/BbsI	MreI/BsaI
**AND logic gate part vectors**							
**Plasmid**	**Part Contains:**	**Details**	**Resistance**	**Copy Number**	**UN Digest Site**	**UN+1 Digest Site**	**UX Digest Site**
pFL_A	U1_HS4_U2	HS4 (derived from constructs in Lienert et al. 2013, Ref. #33 in main text).	Amp	~500	AscI/BspMI	MauBI/BbsI	MreI/BsaI
pFL_B	U2_HSVreporter_U3	6xTAL118bs-spacer_TK_2xCFP_NLS_STOP_BGHterm_HS4 (derived from	Amp	~500	AscI/BspMI	MauBI/BbsI	MreI/BsaI

		constructs in Lienert et al. 2013, Ref. #33 in main text).					
pFL_C1	U3_C-input_U4	CMV-intC-TAL118-a-C2_t2A_mCherry_BGHterm_HS4 (derived from constructs in Lienert et al. 2013, Ref. #33 in main text).	Amp	~500	AscI/BspMI	MauBI/BbsI	MreI/BsaI
pFL_C2	U3_C-input_A_U4	CMV-intC-TAL118-a-C2_BGHterm_HS4 (derived from constructs in Lienert et al. 2013, Ref. #33 in main text).	Amp	~500	AscI/BspMI	MauBI/BbsI	MreI/BsaI
pFL_D1	U4_N-input_U5	CMV-TAL118-a-N2-intN_NLS_BGHterm_HS4 (derived from constructs in Lienert et al. 2013, Ref. #33 in main text).	Amp	~500	AscI/BspMI	MauBI/BbsI	MreI/BsaI
pFL_D2	U4_mutN-input_U5	CMV-mutTAL118-a-N2-intN_NLS_BGHterm_HS4 (derived from constructs in Lienert et al. 2013, Ref. #33 in main text).	Amp	~500	AscI/BspMI	MauBI/BbsI	MreI/BsaI
pFL_D3	U4_N-input_A_U5	CMV-TAL118-a-N2-intN_NLS_t2A_mCherry_BGHterm_HS4 (derived from constructs in Lienert et al. 2013, Ref. #33 in main text).	Amp	~500	AscI/BspMI	MauBI/BbsI	MreI/BsaI
pFL_D4	U4_mutN-input_A_U5	CMV-mutTAL118-a-N2-intN_NLS_t2A_mCherry_BGHterm_HS4 (derived from constructs in Lienert et al. 2013, Ref. #33 in main text).	Amp	~500	AscI/BspMI	MauBI/BbsI	MreI/BsaI
**Plasmids obtained from companies or other labs**							
**Plasmid**	**Contains:**	**From:**	**Resistance**	**Copy Number**	**UN Digest Site**	**UN+1 Digest Site**	**UX Digest Site**
pDHL374	mCherry	Per Malkus, Johan Paulsson Lab	Amp	~1-5	NA	NA	NA
pEGFP	EGFP	Clontech	Amp	~500	NA	NA	NA
pVioA	VioA-His6 in pET24b	Tim Wencewicz, Chris Walsh Lab.	Kan	~40	NA	NA	NA
pVioB	VioB-His6 in pET24b	Tim Wencewicz, Chris Walsh Lab.	Kan	~40	NA	NA	NA
pVioE	VioE-His6 in pET24b	Tim Wencewicz, Chris Walsh Lab.	Kan	~40	NA	NA	NA
pETDuet-1	T7 expression cassette	Novagen	Amp	~40	NA	NA	NA
pETCoco-1	T7 expression cassette	Novagen	Cam	~1 (Amplifiable to 40 with arabinose)	NA	NA	NA

Table S4. Primers used in this study

Designation	Name	Length	Sequence
JT326	Plac.Rev	52	GAT CTC TAG AGG GGA ATT GTT ATC CGC TCA CAA TTC CAC ACA TTA TAC GAG C
JT328	Plac.Fwd.19bpTRC	54	GAC TAT CGA TGC TGT TGA CAA TTA ATC ATC CGG CTC GTA TAA TGT GTG GAA TTG
JT356	BBa_B1006_TOP	63	/5Phos/CTA GCA AAA AAA AAC CCC GCC CCT GAC AGG GCG GGG TTT TTT TTA CTA GTG CGG CCG CCT GCA
JT357	BBa_B1006_BOT	55	/5Phos/GGC GGC CGC ACT AGT AAA AAA AAC CCC GCC CTG TCA GGG GCG GGG TTT TTT TTT G
JT407	ClaIXbaI_740_Top	69	CGA TGC TGT TGA CAA TTA ATC ATC CGG CTC ATA AAA TTT GTG GAA TTG TGA GCG GAT AAC AAT TCC CCT
JT408	ClaIXbaI_740_Bot	71	CTA GAG GGG AAT TGT TAT CCG CTC ACA ATT CCA CAA ATT TTA TGA GCC GGA TGA TTA ATT GTC AAC AGC AT
JT409	ClaIXbaI_199_Top	69	CGA TGC TGT TGA CAA TTA ATC ATC CGG CTC GTA GTG TCT GTG GAA TTG TGA GCG GAT AAC AAT TCC CCT
JT410	ClaIXbaI_199_Bot	71	CTA GAG GGG AAT TGT TAT CCG CTC ACA ATT CCA CAG ACA CTA CGA GCC GGA TGA TTA ATT GTC AAC AGC AT
JT411	ClaIXbaI_48_Top	69	CGA TGC TGT TAC AAC TTA ATC ATC CGG CTC GTA TAA TGT GTG GAA TTG TGA GCG GAT AAC AAT TCC CCT
JT412	ClaIXbaI_48_Bot	71	CTA GAG GGG AAT TGT TAT CCG CTC ACA ATT CCA CAC ATT ATA CGA GCC GGA TGA TTA AGT TGT AAC AGC AT
JT413	ClaIXbaI_12_Top	69	CGA TGC TGT TGG GGC TTA ATC ATC CGG CTC GTA TAA TGT GTG GAA TTG TGA GCG GAT AAC AAT TCC CCT
JT414	ClaIXbaI_12_Bot	71	CTA GAG GGG AAT TGT TAT CCG CTC ACA ATT CCA CAC ATT ATA CGA GCC GGA TGA TTA AGC CCC AAC AGC AT
JT415	ClaIXbaI_3_Top	69	CGA TGC TGT TTT TAA TTA ATC ATC CGG CTC GTA TTG TAT GTG GAA TTG TGA GCG GAT AAC AAT TCC CCT
JT416	ClaIXbaI_3_Bot	71	CTA GAG GGG AAT TGT TAT CCG CTC ACA ATT CCA CAT ACA ATA CGA GCC GGA TGA TTA ATT AAA AAC AGC AT
JT417	ClaIXbaI_0.75_Top	69	CGA TGC TGT TTT GGT TTA ATC ATC CGG CTC CTA CTC TGT GTG GAA TTG TGA GCG GAT AAC AAT TCC CCT
JT418	ClaIXbaI_0.75_Bot	71	CTA GAG GGG AAT TGT TAT CCG CTC ACA ATT CCA CAC AGA GTA GGA GCC GGA TGA TTA AAC CAA AAC AGC AT
JT446	VioB_RE_Fix_TOP	45	/5Phos/AGC TCT CCA GAG AGG CCC TTG AGC ACC ACC ACC ACC ACC ACT GAC
JT447	VioB_RE_Fix_BOT	45	/5Phos/TCG AGT CAG TGG TGG TGG TGG TGG TGC TCA AGG GCC TCT CTG GAG

Table S5. gBlocks used in this study

gBlock Name	Sequence
gBlock_U1UX_A	GACTGCATGCGCTCTTCTTAAGCATTACTCGCATCCATTCTCAGGCTGTCTCGTCTCGTCTCCGCGCGCGGTCTTCCTGGAGATGATCAAACCTAGGCCAGGATA<br>CATAGATTACCACAACTCCGAGCCCTTCCACCCGCCGGCGAGACCTCTAGAGATC
gBlock_U1UX_B	GATCAGATCTGTGAGCGGCCGCATGAAAGCTTGCTCTTCTTAAGCATTACTCGCATCCATTCTCAGGCTGTCTCGTCTCGTCTCCGCGCGCGGTCTTCCTGGAG<br>ATGATCAAACCTAGGCCAGGATACATAGATTACCACAACTCCGAGCCCTTCCACCCGCCGGCGAGACCTCTAGAGTAC
gBlock_U1U2	GACTCAATTGCTCTTCTTAAGCATTACTCGCATCCATTCTCAGGCTGTCTCGTCTCGTCTCCGCCTGCATTAGGATCGATCTCGATCCCGCGAAATTAATACGACT<br>CACTATAGGGGAATTGTGAGCGGATAACAATTCCCCTCTAGAAATAATTTTGTTTAACTTTAAGAAGGAGATATACCATGGGCAGATCTACTAGTAGCAGCCATCA<br>CCATCATCACCACAGCGGATCCGCTAGCTGACTGCAGAGAAGCTTGCGGCCGCCTCGAGTCGCCTAGGCCGCTGAGCAATAACTAGCATAACCCCTTGGGGCC<br>TCTAAACGGGTCTTGAGGGGTTTTTTGCTGAAAACCTCAGGACCGGTGGCTGGGAGTTCGTAGACGGAAACAAACGCAGAATCCAAGCCGCGCGCGGTCTTCG<br>CCAGGATACATAGATTACCACAACTCCGAGCCCTTCCACCCGCCGGCGAGACCCACGTAGTGAGTC
gBlock_U2U3	GACTCAATTGCTCTTCTTAAGGCTGGGAGTTCGTAGACGGAAACAAACGCAGAATCCAAGCCGCCTGCATTAGGATCGATCTCGATCCCGCGAAATTAATACGAC<br>TCACTATAGGGGAATTGTGAGCGGATAACAATTCCCCTCTAGAAATAATTTTGTTTAACTTTAAGAAGGAGATATACCATGGGCAGATCTACTAGTAGCAGCCATC<br>ACCATCATCACCACAGCGGATCCGCTAGCTGACTGCAGAGAAGCTTGCGGCCGCCTCGAGTCGCCTAGGCCGCTGAGCAATAACTAGCATAACCCCTTGGGGC<br>CTCTAAACGGGTCTTGAGGGGTTTTTTGCTGAAAACCTCAGGACCGGTGGCACTGAAGGTCCTCAATCGCACTGGAAACATCAAGGTCGCGCGCGCGGTCTTC<br>GCCAGGATACATAGATTACCACAACTCCGAGCCCTTCCACCCGCCGGCGAGACCCACGTAGTGAGTC
gBlock_U3U4	GACTCAATTGCTCTTCTTAAGGCACTGAAGGTCCTCAATCGCACTGGAAACATCAAGGTCGCGCCTGCATTAGGATCGATCTCGATCCCGCGAAATTAATACGAC<br>TCACTATAGGGGAATTGTGAGCGGATAACAATTCCCCTCTAGAAATAATTTTGTTTAACTTTAAGAAGGAGATATACCATGGGCAGATCTACTAGTAGCAGCCATC<br>ACCATCATCACCACAGCGGATCCGCTAGCTGACTGCAGAGAAGCTTGCGGCCGCCTCGAGTCGCCTAGGCCGCTGAGCAATAACTAGCATAACCCCTTGGGGC<br>CTCTAAACGGGTCTTGAGGGGTTTTTTGCTGAAAACCTCAGGACCGGTGCTGACCTCCTGCCAGCAATAGTAAGACAACACGCAAAGTCCGCGCGCGGTCTTCG<br>CCAGGATACATAGATTACCACAACTCCGAGCCCTTCCACCCGCCGGCGAGACCCACGTAGTGAGTC
gBlock_U4U5	GACTCAATTGCTCTTCTTAAGCTGACCTCCTGCCAGCAATAGTAAGACAACACGCAAAGTCCGCCTGCATTAGGATCGATCTCGATCCCGCGAAATTAATACGAC<br>TCACTATAGGGGAATTGTGAGCGGATAACAATTCCCCTCTAGAAATAATTTTGTTTAACTTTAAGAAGGAGATATACCATGGGCAGATCTACTAGTAGCAGCCATC<br>ACCATCATCACCACAGCGGATCCGCTAGCTGACTGCAGAGAAGCTTGCGGCCGCCTCGAGTCGCCTAGGCCGCTGAGCAATAACTAGCATAACCCCTTGGGGC<br>CTCTAAACGGGTCTTGAGGGGTTTTTTGCTGAAAACCTCAGGACCGGTGGAGCCAACTCCCTTTACAACCTCACTCAAGTCCGTTAGAGCGCGCGCGGTCTTCG<br>CCAGGATACATAGATTACCACAACTCCGAGCCCTTCCACCCGCCGGCGAGACCCACGTAGTGAGTC
gBlock_U1U2_FL	CCCACAGCCAATTGACCTGCATGGCGCGCCCATTACTCGCATCCATTCTCAGGCTGTCTCGTCTCGTCTCCGGAATTCGCGGCCGCTTCTAGAGTGGATCCATC<br>CATGGGTGCTAGCTAAAGCTTAGCTCGAGTGGGTACCGTACCGGTAGAGATCTGAACTAGTAGCGGCCGCTGCAGCCGCTGGGAGTTCGTAGACGGAAACAAA<br>CGCAGAATCCAAGCCGCGCGCGGTCTTCGCCAGGATACATAGATTACCACAACTCCGAGCCCTTCCACCCGCCGGCGAGACCATGCATCCCACAGC
gBlock_U2U3_FL	CCCACAGCCAATTGACCTGCATGGCGCGCCGCTGGGAGTTCGTAGACGGAAACAAACGCAGAATCCAAGCCGGAATTCGCGGCCGCTTCTAGAGTGGATCCAT<br>CCATGGGTGCTAGCTAAAGCTTAGCTCGAGTGGGTACCGTACCGGTAGAGATCTGAACTAGTAGCGGCCGCTGCAGCCGCACTGAAGGTCCTCAATCGCACTG<br>GAAACATCAAGGTCGCGCGCGCGGTCTTCGCCAGGATACATAGATTACCACAACTCCGAGCCCTTCCACCCGCCGGCGAGACCATGCATCCCACAGC
gBlock_U3U4_FL	CCCACAGCCAATTGACCTGCATGGCGCGCCGCACTGAAGGTCCTCAATCGCACTGGAAACATCAAGGTCGCGGAATTCGCGGCCGCTTCTAGAGTGGATCCAT<br>CCATGGGTGCTAGCTAAAGCTTAGCTCGAGTGGGTACCGTACCGGTAGAGATCTGAACTAGTAGCGGCCGCTGCAGCCCTGACCTCCTGCCAGCAATAGTAAG<br>ACAACACGCAAAGTCCGCGCGCGGTCTTCGCCAGGATACATAGATTACCACAACTCCGAGCCCTTCCACCCGCCGGCGAGACCATGCATCCCACAGC
gBlock_U4U5_FL	CCCACAGCCAATTGACCTGCATGGCGCGCCCTGACCTCCTGCCAGCAATAGTAAGACAACACGCAAAGTCCGGAATTCGCGGCCGCTTCTAGAGTGGATCCAT<br>CCATGGGTGCTAGCTAAAGCTTAGCTCGAGTGGGTACCGTACCGGTAGAGATCTGAACTAGTAGCGGCCGCTGCAGCCGAGCCAACTCCCTTTACAACCTCACT<br>CAAGTCCGTTAGAGCGCGCGCGGTCTTCGCCAGGATACATAGATTACCACAACTCCGAGCCCTTCCACCCGCCGGCGAGACCATGCATCCCACAGC
